# Targeting interleukin-13 receptor α2 (IL-13Rα2) for glioblastoma therapy with surface functionalized nanocarriers

**DOI:** 10.1080/10717544.2022.2075986

**Published:** 2022-05-25

**Authors:** Ruijia Liang, Cheng Wu, Shiming Liu, Wenyan Zhao

**Affiliations:** a Department of Neurosurgery, Hangzhou Medical College Affiliated Lin’an People’s Hospital, The First People’s Hospital of Hangzhou Lin’an District, Hangzhou, China; b Department of Neurosurgery, Cancer Center, Zhejiang Provincial People’s Hospital (Affiliated People’s Hospital, Hangzhou Medical College), Hangzhou, China; c Department of General Practice Medicine, Center for General Practice Medicine, Zhejiang Provincial People’s Hospital (Affiliated People’s Hospital, Hangzhou Medical College), Hangzhou, China

**Keywords:** GBM, IL13Rα2, immunotherapy, nanotherapy, functionalized nanocarriers

## Abstract

Despite surgical and therapeutic advances, glioblastoma multiforme (GBM) is among the most fatal primary brain tumor that is aggressive in nature. Patients with GBM have a median lifespan of just 15 months when treated with the current standard of therapy, which includes surgical resection and concomitant chemo-radiotherapy. In recent years, nanotechnology has shown considerable promise in treating a variety of illnesses, and certain nanomaterials have been proven to pass the blood–brain barrier (BBB) and stay in glioblastoma tissues. Recent preclinical research suggests that the diagnosis and treatment of brain tumor is significantly explored through the intervention of nanomaterials that has showed enhanced effect. In order to elicit an antitumor response, it is necessary to retain the therapeutic candidates within glioblastoma tissues and this job is effectively carried out by nanocarrier particularly functionalized nanocarriers. In the arena of neoplastic diseases including GBM have achieved great attention in recent decades. Furthermore, interleukin-13 receptor α chain variant 2 (IL13Rα2) is a highly expressed and studied target in GBM that is lacked by the surrounding environment. The absence of IL13Rα2 in surrounding normal tissues has made it a suitable target in glioblastoma therapy. In this review article, we highlighted the role of IL13Rα2 as a potential target in GBM along with design and fabrication of efficient targeting strategies for IL13Rα2 through surface functionalized nanocarriers.

## 1. Introduction

Glioblastoma multiforme (GBM) is a most frequent, common, and aggressive malignant brain tumor that approximately accounts for 48% central nervous system malignant tumors and 57% glioma (Broekman et al., 2018; Le Rhun et al., 2019; Komori, 2022). The average annual age-adjusted incidence of glioblastoma in the USA is 3.21/100,000 population however varied by sex and age (Tan et al., 2020; Carrano et al., 2021). It is more common in men as compared to women with a mean age of 64 at diagnosis phase (Bush et al., 2017). Glioblastoma is categorized as primary and secondary glioblastoma where the later one from clinical and biological point of views develops in astrocytoma (Ohgaki & Kleihues, 2013). Both primary and secondary glioblastoma exhibit similar histological appearance; however, they are two distinct tumors from different precursors (D’Alessio et al., 2019). Therefore, the secondary glioblastoma must be distinguished from primary glioblastoma because of its long execution of clinical duration (Krolicki et al., 2018). Finding a potential target in glioblastoma treatment, the extensively studied and versatile target is IL-13 receptor α2 (IL13Rα2) and it is attributed to its selective and high expression on glioblastoma (Gatto et al., 2021).

In comparison to normal brain tissues, glioblastoma is overexpressed with certain type of cytokine receptors known as IL13Rα2 that exhibit high affinity for IL-13 (Bhardwaj et al., 2018). The functional significance of IL13Rα2 in glioblastoma is still controversial that needs further investigations; however, poor prognosis and refractory mesenchymal phenotype manifest its expression in glioblastoma (Wang et al., 2018). In addition, literature has shown it as a potential target in glioblastoma treatment (Li et al., 2021; Sterner & Sterner, 2021; Sarfraz & Braselmann, 2022). The IL13Rα2 gene RNA transcripts encode 380 amino acid proteins and offers 30,000 expression binding sites (John et al., 2019). It has been reported that IL13Rα2 overexpression promotes glioblastoma tumor progression and as the malignancy grade of glioblastoma increases the IL13Rα2 expression is promoted accordingly (Brown et al., 2013; Akhavan et al., 2019). The reported overexpression of IL-13Rα2 receptor in GBM specimens ranges from as low as 38% to as high as 78% while the overexpression of its gene is reported in 58% patients (Thaci et al., 2014; Bhardwaj et al., 2018; Zeng et al., 2020). This receptor has been found to be expressed in 83% of pediatric and 58% of adult brain tumors (Kawakami et al., 2004; Brown et al., 2013). The glioma-initiating cells express IL13Rα2 receptor, therefore renders it an essential target for glioma therapy (Brown et al., 2012). In addition glioblastoma, IL13Rα2 blocks the normal apoptosis pathway through signal transducer and activator of transcription (STAT)-6 triggered by IL-13/IL-4 and induces STAT-3 up regulation in glioma (Ou et al., 2021; Vázquez Cervantes et al., 2021). In other aggressive carcinomas such as pancreatic and ovarian cancer, IL13Rα2 through extracellular signal-regulated kinase/activator protein-1 pathway promotes the metastasis and invasion process (Jindal, 2018). Similarly, IL13Rα2 up regulates transforming growth factor β (TGF-β) in immune cells thus promotes progression and tumor immune escape (Mangani et al., 2017).

Glioblastoma is conventionally treated with surgery and it is considered as initial treatment approach; however, it is manifested as non-curative approach and resection is not possible in deep glioblastomas and pediatric population (Garcia et al., 2011; Ryall et al., 2020). Radiotherapy is another conventional approach after surgery which is hindered due to its side effects in the form of its worse consequences on healthy cells as well as its poor penetration to target side (Bastiancich et al., 2021; Li et al., 2022). However, radio-chemotherapy is somehow effective rather than radiation therapy alone (Zur et al., 2020). Similarly, chemotherapy is an alternate conventional therapy choice in glioblastoma but due to short plasma half-lives of chemotherapy drugs, poor water solubility and permeability across the blood–brain barrier and blood–brain–tumor barrier, this approach is less effective for glioblastoma therapy (Warren, 2018; Tang et al., 2019). Due to low efficacy of conventional treatment, modalities have led to failure of conventional treatment strategies and need effective therapy options.

Previously, immunotherapy has shown effective outcomes in the treatment and management of metastatic tumors based on lymphocytic recognition of cancers cells and its consecutive destruction by immune system (Guanghui et al., 2019; Baxevanis et al., 2021). The immunotherapy strategies for the glioblastoma treatment include immune check point inhibition, adoptive transfer of effector lymphocytes and vaccination (Alard et al., 2020; Sangro et al., 2021). Findings from these immunotherapy approaches are unsatisfactory, requiring a thorough reevaluation of clinical data to justify the way for use of these immunotherapy strategies in patients suffering from glioblastoma (Weenink et al., 2020). In addition, immune suppressive microenvironment and low immunogenicity have led to immunotherapy resistance in glioblastoma treatment thus posing this approach less effective for glioblastoma therapy (Goenka et al., 2021). Nanotechnology-based delivery of anti-glioblastoma payloads has shown impressive results in delivering the cargoes to desired target with less off-target effects (Akhter et al., 2021). The major benefits of this sort of delivery system include, low off target effect, resolving the problem of hydrophobic therapeutic agents and enhance the half life deserving therapeutic candidates (Liu et al., 2020; Abadi et al., 2021).

Overall, despite the recent development of glioblastoma multimodality therapies including chemotherapy, radiotherapy, surgery, and immunotherapy pose poor prognosis with rare long time survival. Furthermore, the progressive decline in neurologic functions associated with morbidity along with life quality has devastating impact on patients and concerned associates. In this regard, the surface functionalized nanocarriers for targeting IL13Rα2 receptors in glioblastoma have been investigated that provides certain significant outcomes such as avoid the leakage at permeating barriers, overcome the issue of drug resistance due to heterogenic environment of glioblastoma, mimic the retention time of targeting agent and offer high specificity for the target side. This review article focuses on IL13Rα2 receptors being an exposed target overexpressed in glioblastoma and its respective targeting by nano-immunotherapy and nanotherapy using surface functionalized nanocarriers. The current status and improvement in both glioblastoma treatment strategies targeting IL13Rα2 receptors from available literature have been discussed.

## 2. IL13Rα2-structure and function

Interleukin-13 (IL-13) during normal and pathological conditions particularly cancer plays a versatile role in regulating immune environment and responses as well (Chu et al., 2020; Yuan et al., 2022). It has been reported that IL-13 in majority of cells binds with a low affinity monomer (IL13Rα1) followed by IL4Rα forming a complex heterodimer. Further, the resultant complex results in the activation of transcription factors and signal transducers through release of Janus kinases (Pham et al., 2019). In addition to low affinity receptors, IL-13 also binds to high affinity receptors such as IL13Rα2 in cancer cells (Okamoto et al., 2019). The IL13Rα2 gene RNA transcripts encode 380 amino acid proteins and are expressed in normal cells sparsely (Izuhara et al., 2007). While in glioblastoma multiform, IL13Rα2 offers expression for about 30,000 binding sites (Sharma & Debinski, 2018).

**Figure 1.**
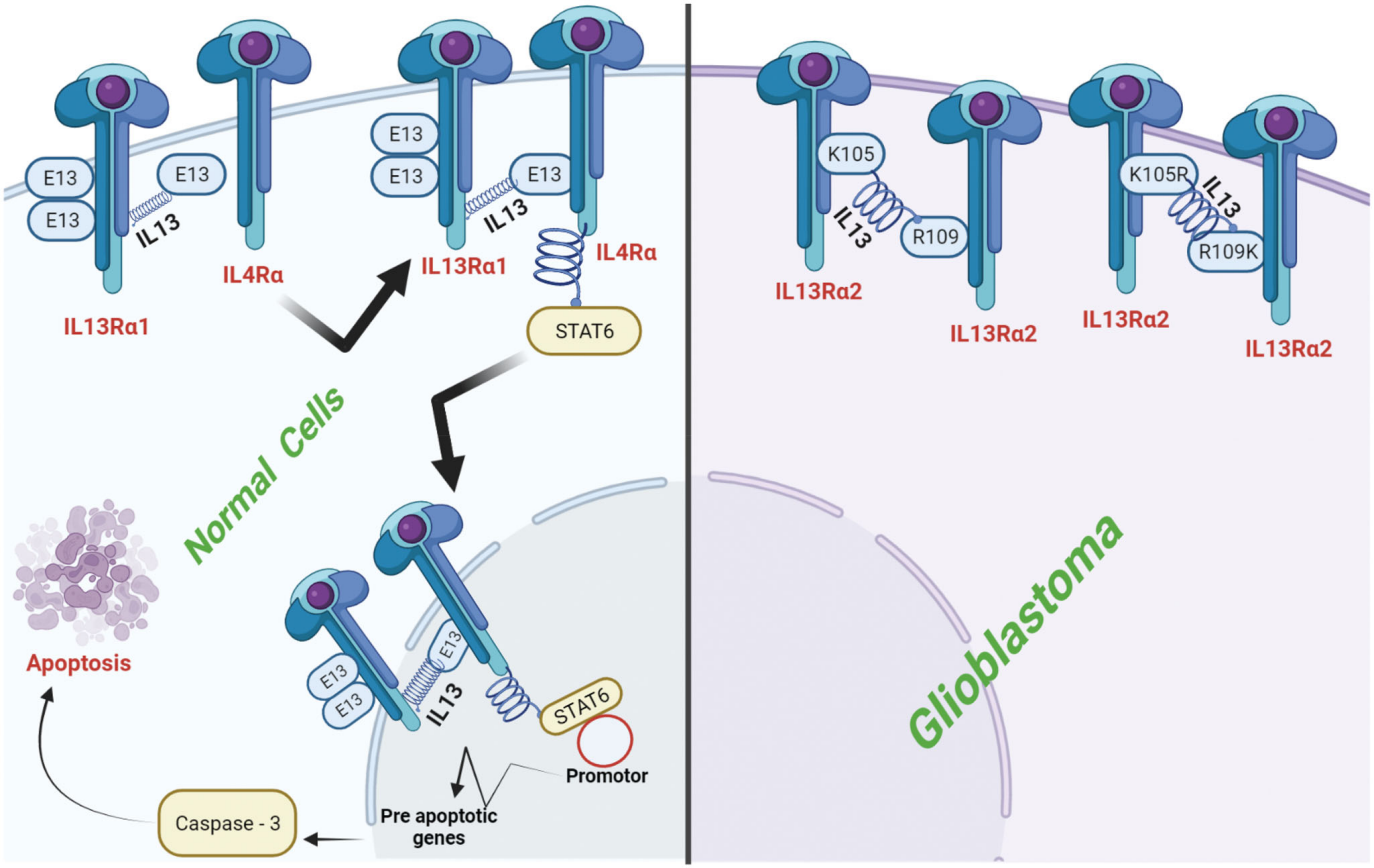
Affinity relationship between IL-13 with IL-13 Rα1 and IL13Rα2. Within normal cells IL-13 using E13 moiety and shows high affinity for IL-13 Rα1 that aids in binding of complex IL-13/IL-13 Rα1 with IL-4Rα that eventually leads to the formation of IL-13/IL-13 Rα1/IL-4Rα complex. Further, the prepared complex activates STAT-6 signaling and translocation to the nucleus takes place. Inside the nucleus, IL-13/IL-13 Rα1/IL-4Rα complex influences apoptotic genes promoters to induce caspase-3 releases that finally end up with apoptosis. On the other hand, within glioblastoma cells R109 and K105 moieties with the help of IL-13 attaches to the interleukin decoy receptors such as IL13Rα2. Such decoy receptor lacks the tail for signaling pathway and thus avoids the abnormal consequences. In association, within glioblastoma cells the IL-13 binding to IL13Rα2 is promoted by mutation in R109 to R109K and K105 to R109K.

The binding of IL13Rα1 to IL-13 in non-mutated cells leads to the activation of STAT-6 signaling that results in translocation to the nucleus (Dey et al., 2020). Inside the nucleus, the activation of resultant pathway regulates the N6-growth arrest specific promoter genes that in turn mimics the activity of caspase-3 and leads to apoptosis (Recio et al., 2019). Similarly, IL13Rα1 blocks IL13Rα2 that further blocks the STAT6 docking to the receptor that eventually impedes activation of apoptosis (Bhardwaj et al., 2018). Such scenario is considered as an alternative mechanism. Furthermore, IL13Rα2 is considered as oncogene due to its selective and high expression in glioma cells along with inhibition of apoptosis subsequently (Chong et al., 2019). The IL-13 ligands as compared to IL13Rα2 binds to IL13Rα1 at multiple cytokine receptor sites. In association, to ensure high affinity attachment the IL13Rα1 requires IL4Rα-heterodimerization. Consequently, these features generate IL-13 ligands with mutated characteristics that exhibit lesser affinity for binding with IL4Rα/IL13Rα1 and greater affinity for binding with IL13Rα2 (Marquardt et al., 2020; Sequeida et al., 2020). On another hand, the mutation in certain locations such as lysine (Lys)-105, 106 and arginine (Arg)-109 either increases or decreases the binding affinity of IL-13 to IL13Rα2 (Hasbum et al., 2021). During scrutiny of such process, high affinity mutants were investigated based on above-mentioned mutations. Moreover, in comparison to native IL-13, the shuffling of Lys to the position of Arg at location 105 resulted in 17 folds high affinity for receptor while replacement of Arg with Lys at location 109 mimicked the receptor affinity by 27 folds (Mrowczynski et al., 2019). Overall, this discussion concludes that it is possible to produce mutants that have low affinity for binding with IL13Rα1 and high affinity for IL13Rα2. The affinity of IL-13 for their decoy receptors is illustrated briefly in Figure 1.

## 3. IL13Rα2 in oncology and glioblastoma

Cancer cells metastatic dissemination affecting distal organs is one of the lethal aspects in patients with breast cancer particularly in basal like breast cancer (Huang et al., 2021). This type of cancer exhibits the potential of metastasis and extravasations from breast to the lungs and is characterized by overexpression of IL13Rα2 genes (Yu et al., 2021). It was reported from a well-characterized cell line model that the expression of IL13Rα2 genes was higher in metastatic cells in comparison with non-metastatic cells that lead to worse metastasis-free survival of patients with high grade tumors (Kalli et al., 2019). In addition, overexpression of IL13Rα2 in advanced cancers has paved way for multiple cancers therapies including colorectal cancer. In this regard, D1 peptide selected from the IL13Rα2 binding site for mouse immunization was evaluated in colorectal cancer. Results showed that blocking of IL13Rα2 signaling treated mice suffering from liver metastasis in colorectal cancer (Jaén et al., 2021). Similarly, another study also proved the over expression of IL13Rα2 in colorectal cancer (Bartolomé et al., 2018). Marquez et al. reported the function of IL13Rα2 in outgrowth of breast cancer brain metastases and investigated IL13Rα2 as a potential target in brain metastasis. It was concluded that at metastatic sites the non-genomic phenotypic adaptations are critical to the progression of brain metastasis and thus IL13Rα2 poses suitable target efficiency for such scenario (Márquez-Ortiz et al., 2021).

In the field of glioblastoma research, IL13Rα2 has been extensively studied and thoroughly investigated tumor specific antigen and glioma specific marker. It was reported that it has up to 75% expression in glioma patients (Sattiraju et al., 2017). In addition, clinical, pre-clinical and *in vitro* studies have shown that IL13Rα2 is a potential target for killing of glioma cells and its targeting by Car-T cells is under clinical trial investigation (Thaci et al., 2014; Brown et al., 2016). In patients with worse prognosis, the expression of these antigens was found more severe and could be possibly explained by certain hypothesis such as, glioma cells adopt overexpression of IL13Rα2 that protect it from immunosuppression (Barderas et al., 2012; Han & Puri, 2018). In association, helper-T cells, mast cells and macrophages trigger the release of IL-13 after activation and it is believed that the produced interleukins then inhibit the pro-inflammatory cytokines and eventually combat inflammatory responses (Yaseen et al., 2020; Zhang et al., 2021). Similarly, IL-13 overexpression was found involved in the immunosuppression. Binding of IL-13Rα1/IL-4Rα and IL-13 results in apoptosis escape in glioblastoma cells usually induced by signal transducer and STAT-6 pathways is considered another significant hypothesis in this case (Han & Kwon, 2018). Conclusively, it is derived that removal of IL-13 from the system would be a factor toward escape of apoptosis from glioblastoma cells.

## 4. Conventional therapies for glioblastoma

Surgery in GBM is the initial way of therapy (Montemurro et al., 2021). Surgery of glioblastoma is related to maximal resection that in turn is associated with overall and progression free survival (Zur et al., 2020). In a broad spectrum, surgery is not patient friendly as well as a non-curative approach and thus patients are exposed further to chemo- and radiotherapy as an adjunct (Zhang et al., 2019). In glioblastoma multimodal primary treatment, radiotherapy takes an integral part (Ziu et al., 2021). A dose of 60 Gy radiations is probably applied to glioblastoma patients (Wegner et al., 2019). Radiotherapy is adopted when surgery is not possible in certain glioblastoma patients (Shirvalilou et al., 2021). However, the effect of radiations on normal cells in association with tumor cells during glioblastoma therapy is a main challenge in the application of this strategy of treatment (Shirvalilou et al., 2021). In addition, radio-chemotherapy is employed in glioblastoma patients for better efficiency (Hsu et al., 2021). To cope with the issue of surgery and radiation therapy, glioblastoma patients are often exposed to chemotherapy.

From chemotherapy point of view, the use of temozolomide – an oral cytotoxic DNA-alkylating chemotherapy agent is now the mainstay of care for the treatment of high-grade glioblastoma (Lajous et al., 2019). In a major phase 3 clinical study, temozolomide with concurrent radiation treatment followed by adjuvant temozolomide for 6 months improved median survival time relative to radiation alone with a twofold improvement in 2-year survival from 10 to 26% (Herrlinger et al., 2019). Glioblastoma patients with MGMT promoter methylation had a better prognosis, with a median overall survival of 46% at 2 years showed in companion research study (Binabaj et al., 2018). However, for the low grade glioblastomas the standard of care is still up for dispute. Low-grade glioma was traditionally treated with external beam radiotherapy. A phase 3 randomized European trial compared early radiation versus observation in low-grade glioma and was allowed only when the tumor progressed; the overall survival was comparable in this study (Reijneveld et al., 2016). The current RTOG study, on the other hand, divided patients with low-grade glioma into risk groups, with high risk patients being those who had residual illness or were over 40 years of age. High-risk patients were randomly assigned to either radiation therapy or radiation therapy with chemotherapy such as procarbazine and vincristine (Shaw et al., 2012; van den Bent, 2014). The overall mean survival rate was improved in patients treated with radiotherapy combined with chemotherapy. However, clinical trials are under investigations to assess chemotherapy alone in high risk low grade glioblastoma patients.

### 4.1. The blood–brain and blood–brain–tumor barriers

Despite the therapeutic potential of chemotherapeutics in glioblastoma, it also exhibits certain limitations in glioblastoma treatment. Drugs that are systemically delivered do not reaches fully to the central nervous system and ultimately to the tumor site (Straehla & Warren, 2020). The greatest challenge in current therapeutic approaches is the decreased delivery of anticancer drugs to tumor site in the brain due to the presence of two important innate barriers, i.e. blood–brain barrier (BBB) and blood–brain–tumor barrier (BBTB). These barriers are protective natural barriers which serve as selective hurdle restricting the entry of most anti-cancer drugs and other xenobiotic to brain (Wang et al., 2019). The BBB yields high electrical resistance of nearly 2000 Ω cm^2^, thus, restricting the entry of drugs to brain (Butt et al., 1990). Additionally, various efflux pumps including P-glycoproteins (P-gps) are present on the surface of BBB that pump-out drugs back to circulation and limit their entry to brain (Qosa et al., 2015). Therefore, nearly all macromolecular substances (i.e. peptides, proteins, and genes) and around 98% of small molecules’ penetration to the brain is inhibited by the BBB (Pardridge, 2007). Neutral, low molecular weight and lipophilic drugs having <500 Da molecular weight can traverse this barrier (Haumann et al., 2020). In real scenario, majority of the anticancer drugs are hydrophilic, charged, and are higher in molecular weight thus are unable to achieve constant and enough concentration in brain (Cao et al., 2020). Effective treatment strategy for brain cancers and GBM needs an efficient delivery system that should be able to penetrate the drug through BBB and accumulate in the brain tumor cells at sufficient concentration for better therapeutic outcome. Various other obstacles including blood–tumor barrier (BTB) and blood–CSF barriers exist that hinder the accumulation of anticancer drugs in brain tumors. In addition, higher interstitial pressure, peritumoral edema and transformed intra-tumoral blood vessels also restrict the delivery of anticancer drugs to brain (Warren, 2018).

The main mechanism through which small hydrophobic drugs traverse BBB is simple diffusion. Additional mechanisms involved in the transport of substances and drugs across the BBB include carrier-mediated transport, diffusion via aqueous porins, active transport and para-cellular transport (Warren, 2018). Additional factors that determine the drugs transport through BBB include the drugs’ physicochemical characteristics, i.e. lipophilicity, drug metabolism, drug clearance, cerebral blood flow, hydrogen bonding, and protein bindings (Banks, 2016). As discussed previously, small sized, highly hydrophobic, less hydrogen bonding, and low molecular weight properties of drugs favor their transport to brain through BBB (Warren, 2018). One way to increase the brain concentration of anticancer drugs is to increase the dose; however, severe side effects of anticancer drugs with higher dose are also challenging (Tang et al., 2021).

Another strategy for increasing the drug penetration through BBB is to transiently disrupt the BBB via biological, chemical, or physical stimuli including surfactants, mannitol, magnetic, and ultrasound heating; however, such approaches are dangerous, unsuitable for most drugs as well as pose high cost (Li et al., 2017). To cope with such issues drug delivery from nanotechnology platform is emerged as versatile delivery system that somehow covers the gap in chemotherapeutic delivery to glioblastoma. However, such delivery system also exhibits certain gaps in the form of retention time at tumor side and deep penetration issue (Hsu et al., 2021). Thus, the use of surface functionalized nanocarriers overcomes such barriers and gives strength to the delivery of therapeutic agents from nanotechnology scaffolds (Yang et al., 2020).

## 5. Targeting strategies for IL13Ra2 in glioblastoma therapy

### 5.1. Immunotherapy

In the arena of glioblastoma therapy, therapeutic antibodies emergence has opened new avenues in the development of novel treatment strategies. In targeting tumor-cell surface specific antigens, the proteins engineering technology plays a versatile role and provide new opportunities (Yoshimura et al., 2014). The conventional methods for payloads delivery have suffered from poor efficiency; therefore, monoclonal antibodies are engineered that effectively deliver the cargo at target side. Such an efficient delivery to tumor related markers have been confirmed in GBM tumors where IL13Rα2 is actively involved (Bhardwaj et al., 2018). It has been confirmed from both *in vitro* and *in vivo studies* that hybridoma cell line development secretes antibodies that are specific and exhibit high affinity for antigen IL13Rα2-associated with tumors and thus provides a novel therapeutic strategy for glioblastoma (Balyasnikova et al., 2012). Remarkably, such antibodies development and its consequent targeting of IL13Rα2 receptors mechanistically block the interaction between IL13 ligand and its cognate receptors. In this context, dendritic cell based therapy has been emerged as potential immunotherapeutic approach due to antibodies availability that have high affinity and specificity for IL13Rα2 (Pituch et al., 2018). On the other hand, within the tumor environment the immune responses associated with anti-tumor activity are suppressed that is overcome by T-cells genetic manipulation and thus such approach can be used as a potential strategy in glioblastoma therapy. In addition, such a T-cells activation triggers the release of cytokines that leads to lysis of IL13Rα2 positive cells (Brown et al., 2018; Pituch et al., 2018).

It is believed that IL13Rα2 is related to poor survival and highly expressed in glioblastoma stem cells (Zheng et al., 2021). Glioblastoma mesenchymal subtype is characterized by proinflammatory nature and is associated with overexpression of IL13Rα2 (Azam et al., 2020). However, normal brain tissue lacks such an over expression (Sai et al., 2017). IL13-zetakine is a complex of IL13Rα2 and Car-T cells that exhibit reduced binding potential for the expression of IL13Rα1/R4α complex (Šamec et al., 2020). Data from phase-I clinical trial have demonstrated that three glioblastoma patients were subjected to collection of peripheral mononuclear cells that were further engineered to CD8+ cytotoxic T lymphocytes expressing IL13-zetakine. During surgery, a Rickham catheter was inserted to the patient and upon recovery T lymphocytes were introduced to the patient. The in-depth observations indicated a significant therapeutic effect with minimal side effects (Brown et al., 2015). Car-T cells therapy in glioblastoma therapy has given birth to serious questions that need response, such as after Car-T therapy, the high immunosuppressive nature needs preservation. Furthermore, glioblastoma within tumor and patients as well exhibit heterogeneous nature that needs focus (Bagley et al., 2018).

**Figure 2.**
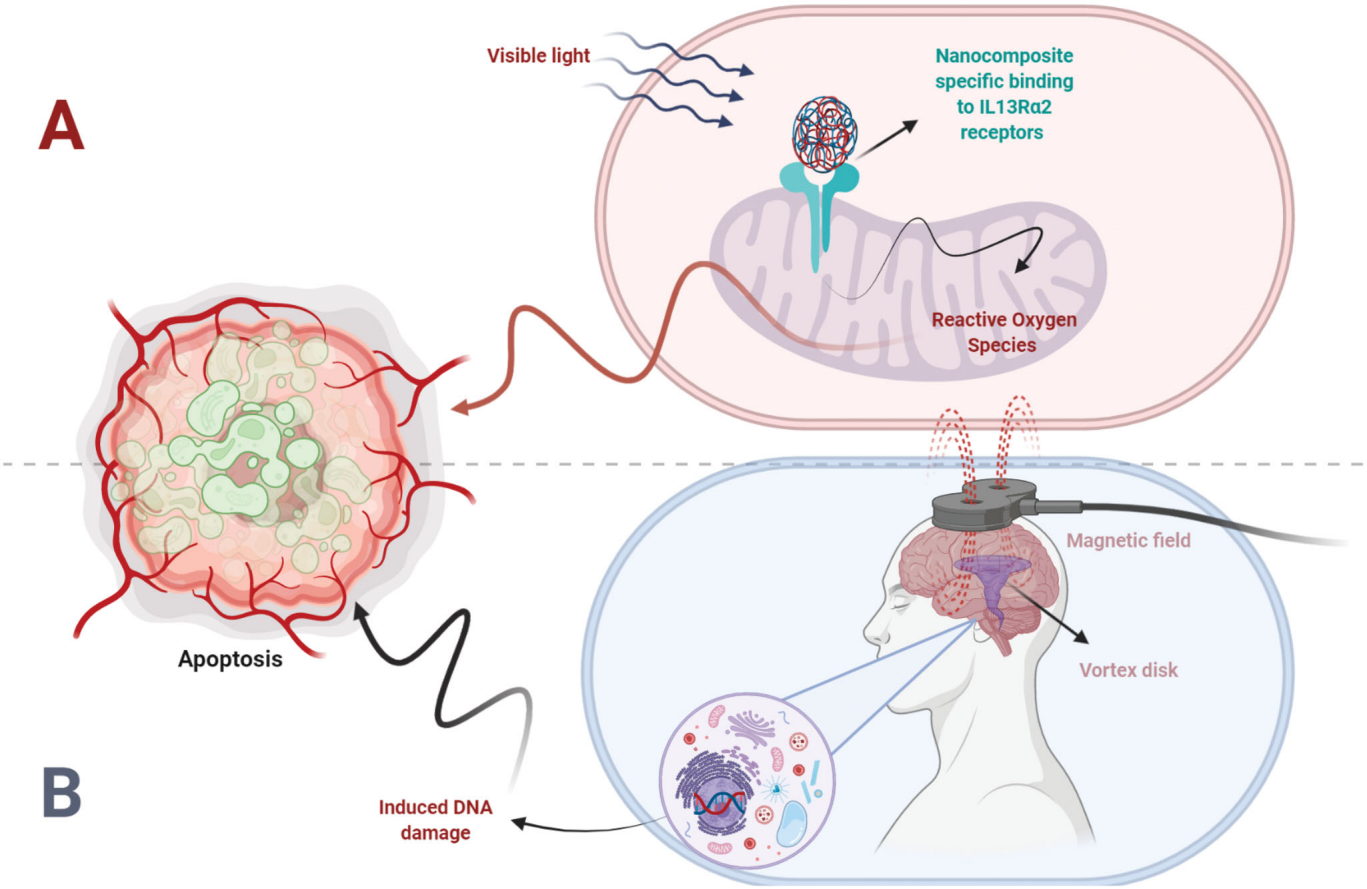
Nano-therapeutics mediated mechanism of apoptosis targeting IL13Rα2 in glioblastoma. The schematic illustration of nanotechnology based therapeutics targeting IL13Rα2 receptors in glioblastoma is shown. (A) The visible applied light to the nanoparticles induces the release of reactive oxygen species that in turn leads to apoptosis. (B) The AC magnetic field applied to the oscillating vortex magnetic disks results in the magnetic nuclear DNA damage that in turn leads to apoptosis.

The nanotechnology-based therapeutics were successfully applied using IL13Rα2 targeting scaffolds, which include vectors based on herpes/adenovirus; TiO_2_-based nanoparticles (NPs), modified T-cells and magnetic vortex disks (Wang et al., 2015; Krenciute et al., 2017). However, it is to worth mention that as compared to IL13Rα2 receptor internalization, IL13Rα2 ablation is better treatment strategy or not, such scenario needs future investigations. Clinical applications of IL13Rα2 targeting platform is briefly elaborated and illustrated in Figure 2. In targeting glioblastoma cells, CAR-T cells modified with a targeted-quadruple-mutant of IL13 was combined with fluorescent therapeutic NPs and targeted to IL13Rα2 overexpressed glioblastoma cells. The NPs were conjugated to the surface of T-cells through azide-alkyne cycloaddition and results showed an eight days retention time for NPs. Doxorubicin loaded NPs conjugated with T-cells showed an improved cytotoxic effect as compared to T-cells alone (Kim et al., 2020). Similarly, in another study, a novel multifunctional glioblastoma targeting nano-drug delivery system was developed using Pep-1 peptide. Pep-1 was used for efficient crossing of BBB and home to glioma cells through IL13Rα2 mediated endocytosis. Results investigated that as compared to non-targeting NPs, the paclitaxel nano-delivery system showed high cellular uptake along high cytotoxicity studies with an IC_50_ value of 0.17 μg/ml in U87MG glioblastoma cell lines. *In vivo* fluorescence imaging showed deep tissue penetration for the fabricated nano-complex system *in vivo* (Wang et al., 2017). In summary, the delivery system to the host cell from immunotherapy platform, chronic host-immune effects and survival in the presence of lymphopenic chemotherapeutic agents are main challenges in the way of immunotherapy. However, the delivery of immunotherapy agents from nano-scaffolds particularly using surface functionalized nanocarriers could be a possible alternative to cope with the mentioned challenges.

### 5.2. Nanotherapy (functionalized nanocarriers)

In the treatment of GBM, one of the reasons for inefficient delivery of therapeutic cargoes at clinical side is drug resistance (Wu et al., 2021). The delivered cargoes for glioma treatment are posed to low efficiency due to glioma tumor heterogenic environment that leads to resistance (Janjua et al., 2021). The genetic diversity in the glioma tumor cell environment induces cell proliferation resulting in tumor reoccurrence before treatment. In association, such tumor reoccurrence is due to the population of resistant cells that avoid the applied therapies from its concerned objective (Malhão et al., 2021). In addition, during progression of glioma, the leakage from BBB is another hurdle in payload delivery (Quader et al., 2022). The structure of BBB poses a major challenge to transport of anticancer drugs into the brain if they are not surface functionalized (Ramalho et al., 2022). Surface functionalization is usually carried out through chemical modification of the nanocarriers’ surface with reactive moieties to provide functional groups that are subsequently conjugated with a targeting ligand of choice. This conjugation of targeting ligand functionalized nanocarriers allows for the selective targeted delivery of the desired therapeutics. Covalent bonding conjugation chemistries have been mostly used for the surface modification of nanocarriers. These covalent strategies include chemical reactions employing reactive carbonyl groups (i.e. reaction of carbonyl groups with alkyoxyamines or hydrazides to form oxime or hydrazine bonds), reactive amine groups (i.e. reaction of amine groups with activated imidoester or carboxylate to form amidine or amide bonds), reactive sulfhydryl groups (e.g. reaction of thiols with gold surface, haloacetyl, maleimide, or pyridyl disulfide or, to form gold-thiol, thioester, or disulfide bonds). Click chemistry has also been used for conjugation of targeting ligand with nanocarrier where azide reacts with alkyne or phosphine to form triazole ring or amide bond (Voigt et al., 2019). Apart from the above covalent reactions, there is one non-covalent interaction which is commonly used for surface modification that is the interaction between biotin and (strept)avidin, the strongest known non-covalent interaction which forms almost irreversible non-covalent binding (Friedman et al., 2013). Such chemical conjugation (covalent or non-covalent) strategies have been used by different researchers in one form or another in the following examples.

Lemasson et al. used IL13Rα2 receptor targeting strategy during pre-clinical evaluation complexed with TiO_2_ NPs. Surface modification of NPs was carried out using dopamine (catecholate linker) that helped in conjugation of NPs with IL13Rα2 antibodies and activation through UV light. The fabricated nano-conjugate system was investigated in A172 glioblastoma cell line. Results showed >80% destruction in glioma cells at a concentration of 6–600 ng/ml. The treated glioma cells were associated with high expression of IL13Rα2 levels (Rozhkova et al., 2009). Antihuman-IL13α2R antibody functionalized magnetic disks were evaluated in N-10 glioma cell lines and it was concluded that IL13α2R serves as a marker for NPs due to its overexpression on glioma cell surface (Kim et al., 2010). It is obvious from literature that IL13α2R is promising target for chemotherapy cytotoxic drugs, i.e. to guide liposomes to the glioblastoma tumor site to cope with overexpressed IL13α2R receptors, doxorubicin loaded liposomes functionalized with IL-13 were fabricated and evaluated. The prepared complex showed better retention of drug at tumor side as compared to free drug along with maximal cytotoxicity. As compared to non-targeted drug liposomes, the surface modified liposomes showed a fivefold reduction in tumor size posing the functionalized nanocarrier as potential alternative in tumor therapy (Madhankumar et al., 2009).

In the domain of targeted drug delivery system, surface functionalized nanocarriers have always shown significant efficacy. In this context, paclitaxel loaded PEGylated NPs were fabricated and functionalized with Pep-1. After evaluation in glioma cell line, the prepared nanoconjugated system showed increased cellular uptake as compared to drug–NPs complex. *In vitro* studies showed IC_50_ of 146 ng/ml for fabricated nanoconjugate system as compared to 349 ng/ml of drug NPs complex. Similarly, enhanced intracranial tumor accumulation of the paclitaxel loaded functionalized NPs was observed that concludes high efficiency and specificity for the glioma tumor cells (Wang et al., 2015). Poor *in vivo* stability and low drug loading capacity are two associated problems with conventional NPs targeting glioblastoma. In this regard, Jiang et al. fabricated paclitaxel loaded self assembled NPs functionalized with Pep-1, glioma homing peptide for targeting glioblastoma through IL13α2R mediated endocytosis. Results showed ultrahigh drug loading capacity of 56.03% and through IL13α2R mediated endocytosis showed high cellular uptake in U87MG cells. The surface functionalized nanocarrier also showed enhanced tumor accumulation and as a whole showed significant antiglioblastoma efficacy (Jiang et al., 2017). In another study, PEGylated polyamidoamine dendrimer NPs functionalized with glioma homing peptides were fabricated and targeted to glioblastoma. Results from *in vitro* and *in vivo* studies indicated higher cellular uptake and efficient tissue penetration for the fabricated surface functionalized nanocarrier system (Jiang et al., 2016). Combined treatment strategy is another aspect of glioblastoma therapy, i.e. antiangiogenesis and anticancer cells. In a research study, to enhance the antiglioma efficacy paclitaxel loaded Pep-1 and CGKRK peptide-modified PEG-PLGA NP were prepared and evaluated. Results indicated long median survival time of 61 days as compared to 34 days of CGKRK-NP-PTX with negligible acute toxicity (Lv et al., 2016). Overall, to evade the hurdles in the way of glioblastoma the surface functionalized nanocarriers pose versatile role in targeting tumor cells in glioblastoma therapy.

Surface modifications particularly through Pep-1 have shown promising results and thanks to its unique role of crossing the blood–brain barrier and blood–brain–tumor barrier. Cilengitide loaded Pep-1 functionalized liposome were designed and evaluated to improve its spatial distribution. The cellular uptake was enhanced to 89% from 47% after surface modification of liposome through peptide. *In vivo* results in U87-bearing xenograft mice showed significant tumor volume reduction (Aronson et al., 2021). To pave the ways for traversing the difficult barriers of blood–brain and blood–brain tumor and target IL13α2R is indeed improved through the discussed surface functionalized nanocarriers. From theranostics platform, in a research study, the fabrication of new functionalized trimetallic nitride template endohedral metallofullerene NPs was reported. The prepared amino acid based nano-platform was conjugated with IL-13 resulting in IL-13 peptide IL-13-Gd3N@C80 (OH) x (NH2) y. The same study concluded that the prepared complex showed enhanced targeting for U-251 GBM cell lines and thus could be used in mice orthotopic models *in vivo* (Li et al., 2015). The cell penetrating peptides have the capability of enhancing the antitumor efficacy; however, the poor selectivity between neoplastic and non-neoplastic cell of penetrating peptides has hampered it from its concerned activity. To achieve enhanced therapeutic efficacy, NPs functionalized with IL-13 were targeted to IL13α2R receptor in glioblastoma tumor that showed mimicked cellular uptake by tumor cells (U87) as compared to endothelial cells. The cellular uptake achieved by functionalized NPs was greater than unmodified NPs. In association, the surface functionalized NPs showed greater penetration effect from vessels to tumor cell with greater rate and speed (Gao et al., 2013). It is concluded that rapid tumor suppression could be achieved using interleukin functionalized nanocarriers for targeting glioblastoma.

In a nutshell, targeting IL13α2R receptor overexpressed in glioblastoma tumors various therapeutic strategies were proposed. It has been shown that IL13α2R targeting moieties were explored for antiglioblastoma effects including anti-IL13R antibodies and ligands due to their high affinity, low immunogenicity, and high biocompatibility. However, the use of functionalized nanocarriers has shown versatile outcomes in the arena of glioblastoma therapy. Such carriers provide specificity and selectivity for targeting IL13α2R receptors in glioblastoma along with efficient penetration into the tumor environment with high rate and speed. It is important to mention that precautionary and safety measure must be ensured in case of those nanocarriers functionalized with hazardous agents such as silica and titanium. Furthermore, targeting IL13Rα2 in glioblastoma using surface functionalized nanocarriers is not fully explored and needs extensive and in-depth investigation in the future.

## 6. Expert opinion

Immunotherapy and nanotherapy using surface functionalized nanocarriers were used recently in glioblastoma therapy to cope with certain hurdles in the way of therapeutic delivery particularly targeting IL13α2R receptors. Actually very limited data are available in this regard and to achieve efficient treatment profiles there is a need for in-depth research studies focusing safety profiles, toxicological profiles as well as clearance mechanisms for surface functionalized nanocarriers targeting IL13α2R receptors in glioblastoma in order to push these strategies toward future advancements. In addition, the fabrication of novel surface functionalized nanocarriers and further exploration of existing carriers will indeed create new opportunities in evaluation of tumor status at particular locations that will lead to the production of conceptualized personal medicines. On the other hand, it is to worth mention that as compared to limited conventional NPs clinical translation, no formulation with surface modified nanocarriers is yet reached to clinical translation side. Thus, clinical translation of functionalized nanocarriers based therapies targeting IL13α2R receptors in glioblastoma will deliberately top up the therapeutic cargoes for glioblastoma from bench top to the clinical arena.

## 7. Conclusions

Glioblastoma multiforme is a lethal and aggressive brain tumor accompanied by low efficacy treatment standards. Surgery, radiation, chemotherapy, and concomitant radio-chemotherapy have been evaluated as treatment options for the treatment and management of glioblastoma. However, low efficacy of these treatment strategies has shown poor therapeutic outcome. Immunotherapy and nanotherapy have shown promising outputs in the treatment of glioblastoma providing long half-lives with sustained release effects to the therapeutic moieties at tumor side. Furthermore, the fabrication of surface functionalized nanocarriers has indeed provided certain unique features from nanotechnology platform including, low off target effects, increased retention time at tumor site, high specificity for the target and enhanced permeation to the glioblastoma tumors. IL13α2R receptors due to absence in the normal surrounding tissues and overexpression at the surface of glioblastoma tissue has made it the most potential target in the treatment of glioblastoma. In recent time, IL13α2R receptors are targeted through therapeutic moieties loaded surface functionalized nanocarriers that have shown a significant therapeutic effect showing it potential therapeutic target for GBM therapy. However, very less literature and studies are available in this regard that need further exploration for the sack of improvement in life quality of glioblastoma patients.
